# I3Mote: An Open Development Platform for the Intelligent Industrial Internet

**DOI:** 10.3390/s17050986

**Published:** 2017-04-28

**Authors:** Borja Martinez, Xavier Vilajosana, Il Han Kim, Jianwei Zhou, Pere Tuset-Peiró, Ariton Xhafa, Dominique Poissonnier, Xiaolin Lu

**Affiliations:** 1 Internet Interdisciplinary Institute (IN3), Open University of Catalonia (UOC), Av. Carl Friedrich Gauss, 5, Castelldefels, Barcelona 08860, Spain; xvilajosana@uoc.edu (X.V.); peretuset@uoc.edu (P.T.-P.); 2 Texas Instruments, Dallas, TX 75243, USA; il-han-kim@ti.com (I.H.K.); j-zhou2@ti.com (J.Z.); axhafa@ti.com (A.X.); d-poissonnier@ti.com (D.P.); xlu@ti.com (X.L.)

**Keywords:** Internet of Things, industry 4.0, I3Mote

## Abstract

In this article we present the Intelligent Industrial Internet (I3) Mote, an open hardware platform targeting industrial connectivity and sensing deployments. The I3Mote features the most advanced low-power components to tackle sensing, on-board computing and wireless/wired connectivity for demanding industrial applications. The platform has been designed to fill the gap in the industrial prototyping and early deployment market with a compact form factor, low-cost and robust industrial design. I3Mote is an advanced and compact prototyping system integrating the required components to be deployed as a product, leveraging the need for adopting industries to build their own tailored solution. This article describes the platform design, firmware and software ecosystem and characterizes its performance in terms of energy consumption.

## 1. Introduction

Nowadays, with the advent of the Industry 4.0, the need for a new generation of monitoring and automation devices has emerged. These devices must cope with the connectivity, complex data processing requirements and energy constraints of novel scenarios, whilst meeting, at the same time, stringent operative costs and industrial-level reliability.

Most of the current industrial devices (e.g., a pump controller or a power meter) are custom-made by manufacturers that integrate different components in a tailored system. Their prototypes are built using different development kits and, when the functionality has been validated, a final product is developed based on the integrated prototypes. This last step is usually time-consuming and costly. The platform-based design approach can provide a solution to meet conflicting design requirements, such as performance, flexibility, design time and cost.

The idea of platform-based design applied to embedded systems is not, of course, new [1,2]. Today, a commonly accepted definition for platform-based design is ”an integration-oriented design approach emphasizing systematic reuse, for developing complex products based upon platforms and compatible hardware and software virtual components, intended to reduce development risks, costs and time-to-market” [3]. This approach has been key, for example, to the vertiginous development and indisputable success of smartphones in recent years.

To support this approach, it is necessary to previously establish the concept of platform. In application-driven design, the term platform is defined as ”a set of subsystems and interfaces developed to form a common structure from which a stream of derivative products can be efficiently developed and produced” [4]. In the definition of platform underlies the concept of “least common multiple”, a commonality among all products of the family. Therefore, the design of a new platform starts from the definition of a set of overall specifications, not for the product itself, but for the application domain.

State-of-the-art automation devices integrate sensing, computing and connectivity capabilities, lately adopting wireless communications. In addition, these devices operate powered by batteries and ideally scavenge energy from the environment to ensure long operational time and hence low maintenance costs. All these elements must form part of the new generation of industrial platforms. Next we outline some of this features:

(a) Wireless communication standards and technologies emerged in industrial process monitoring and automation applications. These standards provided high levels of reliability and low power operation, enabling novel use cases and an important operative cost reduction due to their deployment and maintenance simplicity.
(b) Most industrial processes now being automated require access to different sensors. Moreover, new applications combine the information coming from different types of sensors and use the fused information to perceive the environment more accurately [5]. This trend, called sensor fusion or, more generally, data fusion, results in better and safer decisions when compared to independent systems. Data fusion incurs a computing overhead due to a higher algorithm complexity, which therefore demands higher performance processors.
(c) The low-power requirement has evolved from being a desirable feature to an essential one. Traditionally, the low power consumption has been associated to battery-powered applications, since battery replacement is impractical, often costly and sometimes just impossible. Nowadays low consumption is also a necessity in the industry, even in applications where no battery is required, due to the increasing number of regulations that electronic equipment must meet.
(d) Industrial inter-connectivity, which requires physical attachment to existing equipment.

In this article we present the I3Mote, an ultra-low-power industrial platform designed to meet industrial prototyping and deployment requirements, enabling platform-based product design. The I3Mote platform is targeted at a wide range of industrial applications, including but not limited to: process management, factory automation, field transmitters, industrial wired-wireless bridging and preventive maintenance.

The remaining of the paper is organized as follows. Section 2 introduces, through a historical perspective, different state-of-the-art platforms in the context of industrial prototyping and component-based product design. Then Section 3 states some of the complexities and limitations of today Industrial Internet of Things (IIoT) devices. It also identifies the main barriers that are blocking massive deployment, establishing the I3Mote scope. Section 4 describes the main features of the I3Mote hardware platform. Section 5 introduces the tools and software developed around the I3Mote. Section 6 summarizes the I3Mote performance from a use-case/application point of view. Finally, Section 7 concludes this work with a general discussion about the I3Mote’s key features described in this article.

## 2. Related Work

The number of platforms targeting the Industrial Internet is growing exponentially. Our purpose here is not to present an exhaustive survey. Indeed, we have selected representative platforms with different approaches. These platforms can be classified in many different ways. In this section we have defined three categories, not only according to the solution they provide to end users, but also taking into account the historical perspective.

### 2.1. Sensor-Oriented Platforms

The emergence of microelectromechanical systems (MEMS) sensors in the last decade led to the appearance of their respective development kits. The kits soon incorporated wireless technologies, added different sensors and became advanced development platforms.

One of the most representative platforms of this category is the Texas Instruments SensorTag. The SensorTag is based on the CC2650/CC1350 SoC, which embeds a Cortex-M3 and a 2.4 GHz (CC2650) or Sub-GHz (CC1350) radio transceiver. On the sensors side, the SensorTag include multiple sensors (Infra-red thermopile, 9-axis motion, humidity, pressure, digital micro-phone, magnetic fields and ambient light). On the communications side, the SensorTag supports IEEE 802.15.4 Low-Rate Wireless Personal Area Networks (LR-WPAN) and IEEE 802.15.1 Bluetooth Low Energy (BLE). On the software side a complete solution is provided by Texas Instruments, including RTOS, drivers and multiple application examples.

Recently, Bosch has released the XDK Cross-Domain Development Kit, a platform following a very similar approach. The XDK is also built around a Cortex-M3 microcontroller. As in the SensorTag, the XDK includes multiple sensors (temperature, humidity and pressure, light, sound, acceleration, angular position and magnetic field). Regarding connectivity, the XDK supports IEEE 802.11 (Wi-Fi) and IEEE 802.15.1 (BLE). WiFi support is based on the CC3100 chipset and the TI SimpleLink stack. On the software side, the Bosch XDK platform is based on FreeRTOS.

Both platforms have similar shortcomings. First, there is no interface to any industrial bus, so it is not easy to connect to existing equipment. Second, they focus on sensors on board, but have not been thought to read external sensors that have been already deployed in the industry on a massive scale.

### 2.2. Communication-Oriented Platforms

In the late 1990s, several low-cost wireless technologies were created to fill a gap in the connectivity space, tackling low power communication and addressing machine to machine (M2M) or personal area communications.

Associated with this emergence, a large amount of research activities were developed, which in turn paved the way of a considerably large number of development platforms exploiting unlicensed wireless communications bands. Among them, it is worth mentioning the work developed at the University of California, Berkeley (see [6] for a brief historical introduction to their family of platforms).

An outstanding platform that derived from the UC Berkeley experience was the LTP590x, engineered by Dust Networks (The Smart Dust project at UC Berkeley led to the founding of Dust Networks in 2002, to provide commercial applications of the project. Later on, in 2011, Dust Networks was acquired by Linear Technology, Milpitas, CA, USA). The LTP590x is a platform based on the Eterna SoC (System on Chip), which combines a 32-bit Cortex-M3 microcontroller and an IEEE 802.15.4-compatible radio transceiver. One of the main characteristics of the SoC is the low current consumption of the microcontroller and the radio transceiver. On the communications side, the SoC is compatible with both SmartMesh, WirelessHart (the wireless version of the Highway Addressable Remote Transducer HART protocol) and SmartMesh IP [7]. The LTP590x can be operated autonomously or in combination with an applications processor. When operating autonomously, the LTP590x uses the OnChip software development kit (SDK) to allow users to write applications directly on top of the SmartMesh IP network protocol stack running on the SoC. Contrarily, when operating with an applications processor, the SmartMesh IP network protocol stack runs on the application processor and the SmartMesh C library is used to exchange data with the network processor through a serial port.

Another important milestone in this story was the creation of the OpenWSN project [8], an open-source, standard-based implementation of a complete constrained network protocol stack [9]. Associated with this project, a new generation of open prototyping platforms appeared. OpenMote [6] is the latest Berkeley-designed mote. It is based on a SoC Cortex M3 microcontroller from TI that provides very low energy consumption. The OpenWSN [8] and Contiki [10] projects have been ported to OpenMote complementing an open-hardware and software ecosystem for industrial low-power connectivity. Yet, OpenMote is only addressing network connectivity and embedded sensing without considering industrial device interfacing.

Nowadays, associated with the arrival of the IoT, there is a continuous demand for connectivity in the new generation of devices. This demand has led to a significant increase in the options available in this market segment, both open and proprietary.

A good example is the OpenPicus initiative. OpenPicus is a hardware and software open platform, conceived to speed up the development of professional IoT devices and services. This platform is built around the Flyport concept, a collection of compatible modules with different connectivity technologies (Wi-Fi 802.11g, General Packet Radio Service quadband and Ethernet). The software layer is an open source framework based on FreeRTOS, that manages the TCP/IP software stack, integrated web server and the user application. All the Flyport family members are powered by the same core (the PIC24 low-power processor) and are pin-compatible, which makes them interchangeable, at least at the application level, hiding the type of Internet connection used in each case. This interchangeability feature reinforces the idea of platform-oriented design.

In summary, all these platforms have grown with the idea of providing connectivity to other subsystems (working as a communications module) but do not aim to become the entire product. Some of them have been incorporating new features and sensors, converging towards the sensor platforms discussed in the previous section. Therefore, similar to the sensor platforms described above, they are not intended to connect to existing industrial equipment. Additionally, they lack a complete power management subsystem, which is required to operate as fully independent devices.

### 2.3. Industrial-Oriented Platforms

Unlike in the previous two categories, the variety in the industrial platforms is relatively scarce. Low-power wireless systems have traditionally tried to replace existing equipment, but not to connect to it. This fact has caused that there are few programmable wireless platforms with connectivity to industrial buses.

The Libelium WaspMote [11] platform is one of the exceptions. It is based on a 8-bit ATmega1281 microcontroller. Libelium follows a modular approach, mainly aimed to prototyping purposes. On the sensor side, there are 10 different modules available, with more than 100 sensors in total. In addition, the WaspMote supports several industrial protocols as RS-232, RS-485, Modbus, Controller Area Network (CAN) Bus and 4–20mA. Finally, regarding the communications, the Wasp Mote is based on the Xbee and compatible modules, providing up to 16 radio technologies (Xbee devices have become popular connectivity hardware modules thanks to their well-known form factor and their availability of wide variety of radio technologies, ranging from Zigbee, WiFi and their own proprietary DigiMesh implementation. The devices provide a serial based command interface with a very well-defined set of commands that enable simple technology replacement).

The main problem with this platform is energy consumption. The Wasp Mote is based on a microcontroller literally from the past century, and power management is not up-to-date. A second weak point is its size, a problem derived from its modular approach. A third weak point is its Arduino-based programming model and operating system, limiting the multi-threading capabilities of an application as well as making it impractical for any real time operation.

One interesting initiative that is emerging in recent years is the MangOH project [12] MangOH is an open-source hardware sensor-to-cloud platform for building wireless IoT solutions. The design philosophy behind the MangOH project is simple: MangOH designs try to cover 90% of the IoT prototypes out-of-the-box so that software developers can easily create reliable solutions and hardware designers can customize it to create new hardware IoT devices in a short time.

The MangOH ecosystem is built around the Green platform, an embedded Linux board with different connectivity options (USB, RS232, RJ45...) and multiple expansion slots. There are several expansion modules currently available. Among them, it is worth mentioning the industrial Ethernet and the CAN bus cards, which provides industrial connectivity.

As in the case of Libelium, this platform is not targeted at high efficiency and low energy consumption, but modularity and ease of development. Therefore, and still being an interesting alternative, it does not cover the requirements addressed in this work.

In Table 1, we have summarized the review in this section according to four criteria: low-power capabilities, wireless standards, industrial connectivity and sensing capabilities.

In summary, the studied platforms lack one or other features that we believe are needed to support platform-based design for industrial products, as presented in Section 1. First, power management for battery operation and support for harvesting technologies should be integrated in the platform, but it has rarely been done. Second, industrial wireless connectivity is partially supported as some of the technologies are too power hungry or not reliable enough for certain scenarios. Third, they lack support and interfaces to external sensors that are already present in the industry. Finally, we have seen almost none of them providing the capability to interface to industrial buses, with the exception of those platforms with a clear orientation to prototyping.

## 3. Problem Statement and I3Mote Scope

The IoT is still not a reality in the industry. While there is a non-questionable interest to adopt new solutions for that long-awaited transformation, there are still barriers that keep the industry in a cautious position. In the past we have already seen good technology hypes which have not been materialized. In order to avoid this situation, it is time now for all involved stakeholders to analyze the current industrial needs in order to enable the new technology to position itself where is needed. Now, with some historical perspective, we identify below several weaknesses that are slowing down the adoption of IoT technology in the industry.

First, the technology must become more accessible, attractive and, above all, easy to integrate. Only when embedding the Internet of Things becomes as simple as using a smartphone application, the industry will adopt this technology in a massive way. For this purpose, an important requirement is to be compliant with industrial standards. In particular, communication’s standardization is essential to lower the entry barriers for the industry, and to improve the interoperability of different applications/systems [13].

Second, industry needs end-to-end solutions, not partial ones. Designers of new platforms should take special care to cover complete use cases, not just a part of them. As seen from the related work review, there are many development kits that have grown around a particular device, but few platforms have been designed as a full solution.

Most notably, wireless platforms and industrial equipment seem to have evolved in parallel, but not jointly. Somehow, we have always had in mind to replace existing equipment (wireless as an alternative) but not to build on top of the existing equipment (wireless as added value). In terms of hardware, this has resulted in a fundamental deficiency in the wireless platforms, namely the ability to connect new platforms to the industrial equipment already in operation.

Third, key accelerators for the industry adoption are open-source hardware and software projects. The experience has taught us several lessons [6]. First, tight coupling and parallel evolution between hardware platforms and open-source projects benefit the adoption of the standards they implement. Second, providing easy-to-use board support packages (BSP) and prototyping tools speeds up time-to-deployment and eventually time-to-market. Third, open hardware benefits knowledge transfer and industrial adoption, as companies can take advantage of already proven designs. Last, symbiotic alignment between standardization groups and open-source hardware/software projects yields better standards and speeds up their adoption.

The I3Mote platform has been designed to provide solutions to the aforementioned problems. This approach is based on the three following cornerstones:

### 3.1. Full Product Coverage

**Figure 1:**
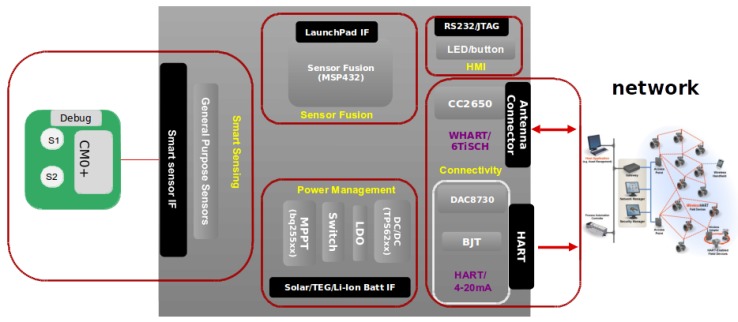
I3Mote platform.

I3Mote integrates most of the components needed to be deployed as a product, avoiding in some cases the need to build a custom solution. Being specifically designed for this purpose, the I3Mote features the most advanced low power components to cover industrial sensing, on board computing and industrial wireless/wired connectivity, in an attempt to cover all the specifications and weakness described in Section 1 and Section 2 (see Table 1). In short (see Figure 1):

- The I3Mote features a multi-source power solution. It supports energy harvesting from thermoelectric or solar/indoor light sources. Energy can be stored in rechargeable batteries or just drawn from primary batteries. It also supports the industrial grade power lines over 8–36 V, being essential when there is already available power in an existing industrial network. For maximum flexibility, the platform is designed to provide full software control over different power management options.
- Sensor fusion is built around a high performance, ultra-low-power Advanced RISC Machine (ARM) Cortex-M4F core, the MSP432. The platform includes several on-board sensors (temperature, humidity, acceleration, ambient light, pressure) as well as an Smart Sensor Interface (SSI), a “de-facto” standard to enable multimodal extension.
- Communications rely on an advanced low power wireless SoC, the CC2650, featuring an ARM Cortex-M3 core and a 2.4 GHz-compliant radio able to support IEEE 802.15.4 on 2.4 GHz, Bluetooth and BLE. It is also perfectly suited for the new wave of industrial Internet of things (IIoT) deterministic standards such as IEEE 802.15.4-2015 time-slotted channel hopping (TSCH) and the IETF 6TiSCH stack.
- Thanks to its Highway Addressable Remote Transducer (HART) interface, the I3Mote provides a perfect solution for a wired-wireless bridge, wireless redundancy of a wired-channel, HART sensor wireless replacement and the existing 4–20 mA/HART connectivity.

This deployment-oriented approach is reinforced by a robust design to meet the industrial-grade reliability.

### 3.2. A Complete Ecosystem

**Figure 2:**
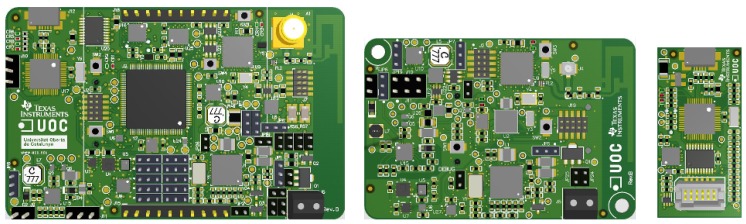
I3mote platform versions: ”Engineering” (**left**), ”Tiny” (**center**) and ”Daughter” (**right**).

The I3Mote is an open hardware platform aimed to fill the gap between the prototyping and early deployment market. In essence, the idea is to provide different form factors (Figure 2) with the same functionality and pin-to-pin compatibility: first, an “Engineering” version (left), whose aim is to facilitate in-lab prototyping, and second, a compact and integrated version (center) devoted to field evaluation and deployment. The functionality of the “Tiny” board is extended with the “Daughter” board (right), which basically provides the debugging capabilities already available in the “Engineering” version. This feature facilitates the transition from the prototype to the product.

The hardware ecosystem is complemented by a suite of software tools and ports to popular open-source IoT implementations. This out-of-the-box software package enables the use of I3Mote as reference design to quickly set up a wireless, wired or hybrid (both wireless and wired) network that performs actuation and data extraction from various types of sensors.

An additional feature towards ease of use is the separation between the application processor and the communications processor. In a very similar way to Dust Networks module, the CC2650 can operate autonomously, allowing users to write applications directly on top of the network protocol stack. More interestingly, when operating with the applications processor (MSP432), the network protocol stack runs in the CC2650 and the application processor exchanges data with the network processor through a serial port. In this way, the application is completely decoupled from communications (to such an extent that the stack of communications can be supplied in binary form), significantly simplifying the development. Notably, the features of the CC2650 allows for easy exchange of the communications standard, following a similar approach to the OpenPicus platform. However, in this case it is not necessary to change the HW module, but simply the SW stack running on the CC2650.

### 3.3. Open Hardware

The I3Mote design is licensed under a non-viral CERN 2 OHL open hardware license, enabling the extension, modification or integration of the features of this platform to industrial developments without interfering with the licensing of the industrial product.

In this way, although the I3Mote is designed to completely cover a large number of use cases, hardware engineers can extend (or prune) features customized to their own needs in a short time and with lower risk, following the same principles as other open platforms such as MangoH or OpenMote.

## 4. Platform Hardware

**Figure 3:**
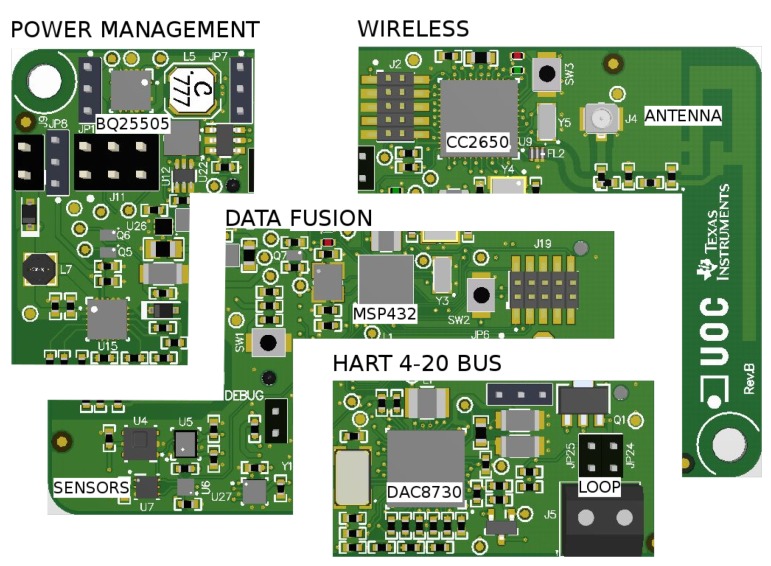
I3Mote blocks.

The I3Mote is designed to cover industrial sensing, on board computing (e.g., sensor/data fusion applications) and industrial wireless/wired connectivity. These functionality requirements determine the partition of the main HW blocks and their placement on the Printed Circuit Board (PCB). The design is oriented towards robustness to meet industrial reliability requirements, while respecting a compact form factor to allow integration in industrial equipments. General partitioning of the hardware can be shown in Figure 3:

- Multi-Source Energy Management Unit: power sources can be CR2032 coin-cell batteries (on-board), AA and AAA standard batteries (external connector), multi-source energy harvesting (e.g., thermoelectric or solar/indoor light sources), or 24–36 V industrial-grade DC power line. In addition, the I3Mote can be powered by an external 5 V source, like an USB cable. USB support is embedded in the “Engineering” version, and provided by the Daughter board for the “Tiny” one, as shown in Figure 2.
- Data Fusion Unit: featuring a dedicated high performance processor, built-in sensors (temperature, humidity, acceleration, ambient light, pressure) and Smart Sensor Interface (SSI) to enable multimodal extension.
- Industrial-Grade Wireless Connectivity: build around an advanced multi-standard wireless SOC compliant with, among others, the IEEE 802.15.4-2015 and with a PCB or an external antenna.
- Industrial Wired Connectivity: the I3Mote integrates all components required for loop-powered sensor transmitter (4–20 mA) and HART modem.

In this section, we go through each part in detail.

### 4.1. Power Management

**Figure 4:**
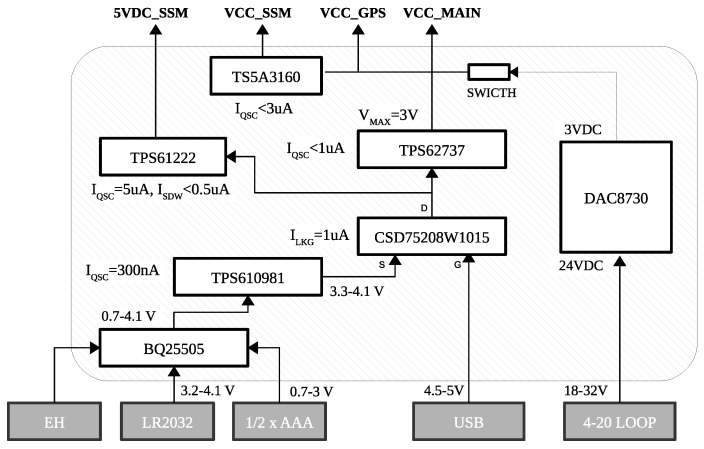
I3Mote power distribution.

The I3Mote integrates a power management unit (see Figure 4) capable of collecting energy from thermoelectric, solar and other harvesting sources. The energy can be stored into a large capacitor reservoir (through an external connector) or a secondary cell (i.e., a rechargeable battery).

Once there is enough charge stored in that bank the system can be powered from the reservoir. Alternatively, if sufficient charge has not been collected, a backup non-rechargeable battery (e.g., AA or AAA) can provide power to the system. The power management device gives an indication of the actively used power source (harvesting cells or battery) to the microcontroller unit (MCU) by using a general purpose input/output (GPIO) pin.

There are two main voltages in the system: 3 V and 5 V. The 3 V (VCC_MAIN) source is used for MCUs, sensors, switches, etc. and the 5 V is only used for external logic. The 3 V is driven by two DC/DC stages. The first stage relies on a boost/bypass DC/DC converter (TPS610981) that is capable of raising the voltage to 3.3 V from voltages as low as 0.7 V. Remarkably, this allows to power the system from a single alkaline cell. The second stage is driven by an ultra-low power DC/DC converter (TPS62737) which generates the 3 V voltage for the main power domain. This buck converter accepts input voltages up to 5.5 V, which allows the use of 5 V power supplies for the I3Mote (e.g., USB). A P-FET based device (CSD75208) protects the battery from this 5 V source as well as power or-ring, i.e., if the USB power is connected then the battery power is not drained. From the power management unit, it is worth mentioning the following components:

- *Energy Harvesting Manager IC:* The BQ25505 regulates the energy provided by the energy harvesting cells. The BQ25505 is specifically designed to efficiently extract microwatt to milliwatt of power generated from high-impedance sources, as solar cells, using maximum power point tracking (MPPT). The harvested power is stored in a ceramic super-capacitor reservoir or a rechargeable battery. The BQ25505 also manages a backup battery, which is used when there is not enough ambient energy to power the sensor node. Furthermore, by changing the values of three external resistors, developers can set the voltage levels of the energy reservoir that allow the BQ25505 to properly connect and disconnect the two power sources to the load. The state of the battery is monitored via the VBAT_OK line. By means of this line, the BQ25505 indicates to the system processors if the rechargeable battery is between the programmed load levels or, on the contrary, the power manager has switched to operate from the backup battery. In this way, the application can make the appropriate decisions accordingly. Developers can also program the MPPT sampling network to optimize the input power provided by the solar cell.
- *Primary and Secondary Batteries:* An embedded CR2032 cell battery can be used as a backup battery for the continual sensing operation. This battery type was chosen due to its ubiquity and its small size. The on-board battery holder can be used both as backup non-rechargeable battery (primary) and as the rechargeable battery (secondary). In addition, the I3Mote incorporates standard interfaces for connecting larger external batteries, both primary and non-rechargeable.
- *Power Switches Control:* The I3Mote provides software control to independently switch on/off different blocks of the circuit. For this purpose, the I3Mote includes a TS5A3160 single-pole double-throw (SPDT) analog switch. The switch enables the SSI 3 V output. Additionally, there is a specific boost converter (TPS61222) to enable 5 V at the SSI. Both 3 V and 5 V sources are designed to provide a clean power to external components (e.g., sensors).

### 4.2. Data Fusion

**Figure 5:**
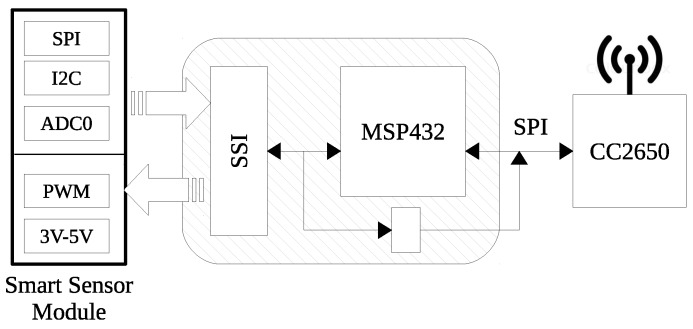
Data fusion architecture.

The platform is equipped with a high performance processor with high-computing capabilities. This enables the I3Mote not only to collect data from multiple sensors, but to manage the data received beyond the simple preprocessing (e.g., aggregation, compression, mining, etc.) The board includes an expansion socket that allows the connection of external sensing systems (e.g., industrial probes). The module is complemented by several on-board sensors with multiple functions, as well as all the required connectivity. In more detail, the data-fusion unit (Figure 5) includes the following modules:

- MSP432: The MSP432 is the data fusion core. This advanced MCU platform is built around the high performance ARM Cortex-M4F core, featuring DSP extensions and an integrated FPU (Floating-Point Unit). The MSP432 acts as the data hub of the board. This involves having full access to the different sources of data, mainly on-board and external sensors, with direct access to the SSI. The MSP342 is connected to the communications processor (CC2650) through a dedicated high-speed Serial Peripheral Interface (SPI).
- Non-Volatile Memory: The board integrates a 32 K×8 (256 Kbit) Serial Electrically Erasable PROM (EEPROM). This device is capable of both random and sequential reads up to the 256 K boundary. The 24LC256 has a 2-wire inter-integrated circuit (I2C) compatible serial interface accessible from both the MSP432 and the CC2650. Therefore, it can be used as a mail-box to exchange messages between the two processors, as an off-line alternative to the dedicated SPI channel.
- Smart Sensor Interface: The SSI is a 2 × 10 pin, 1.27 mm expansion header designed to connect to an application-specific module. The SSI includes a standard SPI port enhanced with an interrupt line, I2C, 2 pulse with modulation (PWM) pins, 2 single analog to digital converter (ADC) lines or 1 differential pair, 4–20 mA bus lines and 3/5 V power sources.
- On Board Sensors: The I3Mote incorporates multiple on-board sensors. The specific ICs belong to the new generation of high-performance low-cost sensors including: an ambient light sensor (OPT3001), a temperature/humidity sensor (HDC1080), a MEMS thermopile sensor (TMP007) for contactless temperature measurements, an absolute barometric pressure MEMS sensor (BMP280) and, finally, a three-axis linear accelerometer (LIS2HH12). All these devices have been selected among those with the lowest power consumption on the market.

### 4.3. Wireless Connectivity

**Figure 6:**
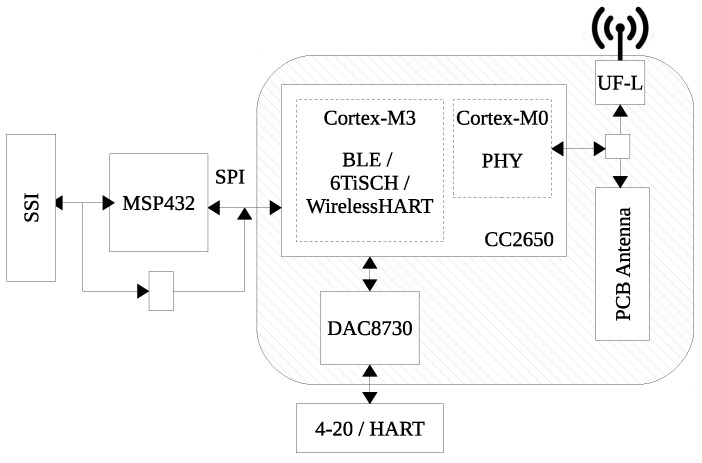
I3Mote communications.

The wireless connectivity unit (see Figure 6) of the I3Mote is built around the CC2650, a multi-standard 2.4 GHz ultra-low power MCU. The CC2650 is designed to integrate the complete stack of standard protocols such as Bluetooth and 6LoWPAN (IPv6 over Low Power Wireless Personal Area Networks), enabling seamless connectivity for industrial, consumer electronics and medical applications. The CC2650 embeds three different processors: application, radio and a dedicated sensor acquisition unit. The goal of this SoC architecture is to maximize performance while reducing power consumption.

The radio controller is built around a 32-bit ARM Cortex-M0 processor. It is responsible for interfacing to the radio hardware and running the PHY layer of the protocol stack (both for IEEE 802.15.4 and BLE). The application processor is a 32-bit ARM Cortex-M3 running at up to 48 MHz. It is designed for higher-level protocol and application layers processing. The sensor controller is built around an ultra-low-power processor and is used to interface external sensors and collect analog/digital data autonomously while the rest of the system is in sleep mode. This module can be used as an alternative to the MSP432 when data processing is not necessary. In addition, the CC2650 includes various communication and cryptographic peripherals.

The I3Mote does not require external components for wireless radio communications. For this purpose, it includes a PCB Inverted F Antenna and all required matching components. The antenna is designed to be used with 2.4 GHz transmitters and receivers. The maximum gain is measured to be +3.3 dB and overall size is 25.7 × 7.5 mm${}^{2}$. Alternatively, the I3Mote contains a 50 Ω U-FL connector to use with any standard 2.4 GHz external antenna.

### 4.4. Wired Communications (4–20 mA/HART)

The I3Mote platform uses the DAC8730 IC, which integrates several key components for a loop-powered sensor transmitter including HART and Foundation Fieldbus modems, 16-bit PWM Digital-to-Analog Converter (DAC), voltage-to-current converter, reference voltage and regulator. This chip operates over 24–36 V industrial-grade power line. The 16-bit PWM DAC and modem outputs are filtered externally in order to generate the respective DC and AC signals for the smart loop powered transmitter. These signals are then fed back to the internal operational amplifiers and other internal circuitry along with a few external components to control the loop current. The 16-bit PWM DAC data and the HART data are controlled via SPI and UART interfaces from the CC2650.

Additionally, the internal buck converter generates a 3 V supply that can be used to power external components such as MCUs, sensors, etc. The I3Mote board is designed to take advantage of this feature, i.e., the 24–36 V line can be used as power source for the whole system (See Figure 4). The amount of current that DAC8730 can provide to the system is up to 95 mA assuming 24 mA setting is used over the line.

### 4.5. Programming and debug

The I3Mote board features a JTAG interface for programming and debugging the two MCUs included on the board, the CC2650 and the MSP432. In addition, both processors can be programmed making use of a mechanism known as Bootstrap Loader (BSL), a built-in application that allows programming through a serial interface. In addition, the I3Mote includes a USB-serial bridge, which allows in-field programming with pre-compiled images and receiving status information through USB. This IC is present in the “Engineering” version, and it is provided by the daughter board for the “Tiny” version (See Figure 2).

## 5. Software and Protocol Stack

The I3Mote has been designed to meet industrial level requirements in terms of performance, interfacing to industry devices and standards. This design has also taken the software and communication stack into account. This section describes the software ecosystem supporting the platform.

### 5.1. Software Tools

A hardware platform needs to be accompanied by a set of development tools that facilitate its use, specially in the prototyping space where adopters need to quickly startup the platform. The I3Mote software ecosystem is built through a set of license-free tools including:

- Toolchain: A license-free toolchain is offered through the Code Composer Studio (since version 7), an integrated development environment (IDE) based in Eclipse and open-sourced by TI. The toolchain comes with different configurations supporting the GNU ARM GCC compiler and an optimized version referred as TI ARM Compiler. The IDE provides debugging capabilities through different JTAG/SWD devices and supports all TI embedded microcontrollers, in particular the CC26XX and the MSP43X families.
- Real Time Operating System (RTOS): the provided tools include the TI-RTOS, a real-time operating system specially tailored to TI low-power microcontrollers. TI-RTOS provides task abstractions and a priority-based preemptive scheduler which enables real-time operation and ensures extremely low-power operation of the platform, thanks to its tight integration with the TI device abstraction layers. In addition, I3Mote has been ported to other real time operating systems such as FreeRTOS and non-preemptive operating systems such as Contiki (only the CC2650).
- Gateway: The mesh network built using the TI 6TiSCH stack is rooted at a gateway device, acting as the 6LoWPAN Low Power Border Router (6LBR) of the network. Its functionalities range from 6LoWPAN to IPv6 packet inflation to the possibility to run a centralized scheduling mechanism for the network. The gateway software runs on a BeagleBone Black [14] device attached to an I3Mote that acts as the L2 network interface of the mesh network. The I3Mote uses a serial interface to bridge packets to the 6LBR. From the 6LBR IPv6 packets are forwarded to the Internet.
- Backend Demo: A backend demo graphical user interface (GUI) developed with Node.js is also part of the open tools provided with the board. This back-end software can be executed in any cloud instance or server and used to monitor the status of the nodes in the network as well as to gather sensor information through IETF CoAP [15].

### 5.2. Protocol Stack

The I3Mote network processor is equipped with an IEEE 802.15.4 and BLE communication interface as described in the previous section. The software ecosystem developed to support the board integrates an industrial-grade protocol stack implementing a set of standards developed by the IEEE and the IETF. These set of protocols are referred as the IETF 6TiSCH stack [16]. 6TiSCH is rooted at the Std. IEEE 802.15.4 TSCH Medium Access Control (MAC) that provides deterministic access enabling industrial-grade reliability [17] and ultra-low power operation [18]. On top, this set of standards being defined by the IETF 6TiSCH working group provide network bootstrapping, security bootstrapping and distributed scheduling functionalities [19] and policies [20,21]. In addition, this standardized protocol stack uses the IETF 6LoWPAN and Routing Protocol for Low Power Lossy Networks (RPL) [22] to achieve IPv6-based end-to-end connectivity while meeting the determinism, robustness and low-power operation required for battery-operated devices targeted at industrial process monitoring and control applications [9].

**Figure 7:**
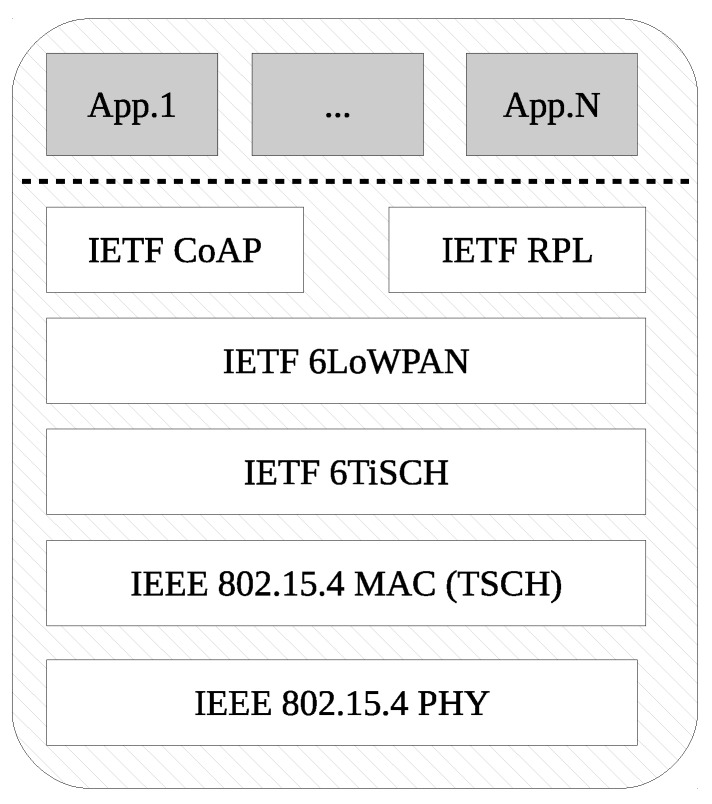
6TiSCH protocol stack for end device.

In particular, the 6TiSCH protocol stack implemented (See Figure 7) enables highly reliable communication and optimal low-power operation [23]. This is achieved thanks to a TSCH MAC layer. This MAC layer divides time in slots for each of the available frequencies. This matrix is seen as a scheduler in time and frequency where nodes in the network are allocated certain cells by a scheduling mechanism. The bandwidth, latency, energy consumption and reliability can be traded off through different schedule configurations enabling network flexibility and supporting, at the same time, collision-free communications.

It is worth noting that IPv6 connectivity, mesh routing through RPL and transport protocols, such as User Datagram Protocol (UDP) and CoAP, are exposed to applications running on the data fusion processor through a serial application programming interface (API). This ensures a clear separation of the network core and the data processing unit, and simplifies prototyping and product development by offering connectivity as a service.

### 5.3. IPv6 Low-Power Border Router (6LBR) Architecture

**Figure 8:**
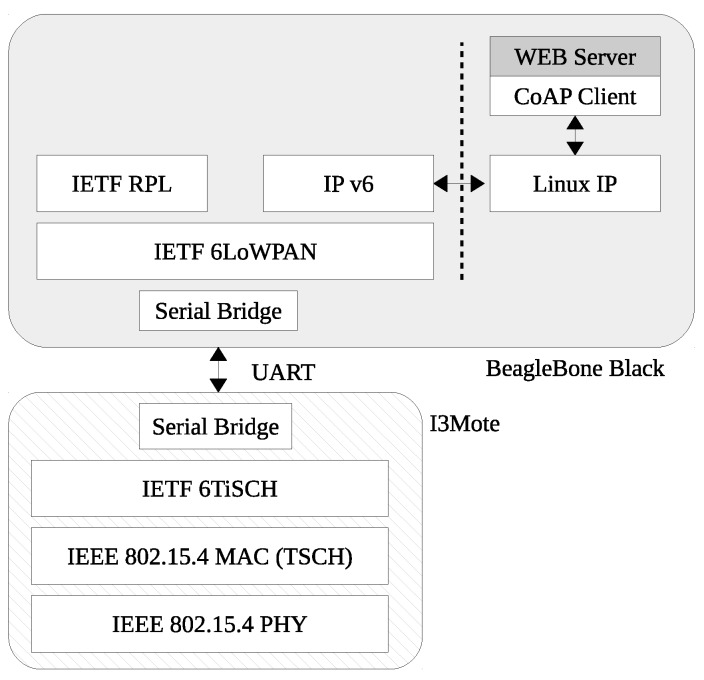
6LBR architecture.

The Gateway (6LBR) architecture is depicted in Figure 8. An I3Mote is connected directly to a Linux-capable device such as BeagleBone Black, and acts as a layer 2 bridge. Incoming radio packets are handled by the TSCH MAC, passed through the IETF 6TiSCH layer and forwarded through the serial interface to the Linux-capable device. The 6LBR software runs as an O.S. daemon and is in charge of the 6LoWPAN decompression to IPv6. It also handles routing information such as the RPL state and source routes. Once an IPv6 packet is decompressed from 6LoWPAN this is injected to the Linux IP stack through a TUN interface that forwards it to a CoAP Client running as a service. The CoAP Client then injects the obtained data to a database through a web server API. Downstream, the process is similar, the CoAP client issues a message that is forwarded through the TUN interface to reach the 6LBR that compresses the IPv6 packet to 6LoWPAN, appends the required routing information (e.g., source routing header from RPL) and forwards it to the serial interface. At the I3Mote, the packet is received from the serial interface and queued for transmission by the 6TiSCH layer.

### 5.4. Licensing

The I3Mote ecosystem is composed of multiple elements subject to several licensing types. Most of the tools such as IDE, compiler, TI-RTOS and software examples have been recently released following different open-source licensing formats. In the case of the network processor protocol stack, various alternatives can be found, ranging from proprietary licensed libraries implementing WirelessHART, the IETF 6TiSCH stack with a mix of open-source and licensed components and fully open-source implementation supported by community projects such as Contiki. In particular, 6TiSCH stack provided today by TI is an exemplary software stack for hardware evaluation. Other software stacks such as BLE, ZigBee, etc. can be utilized for hardware evaluation as well.

## 6. Performance Evaluation

This section presents a non-exhaustive analysis of several performance results measured on the I3Mote. We focus on highlighting experimental results through a representative use case based on a periodic sampling/reporting application.

Basically, in a periodic reporting application, the device wakes up periodically and puts the sensors in the active state, waiting the required stabilization time. After reading the sensors, the device reports the values to a central node through a wireless link and then goes back to the sleep or idle state. This example involves three key blocks of the I3Mote, which we address in this section: (1) low-power wireless communication using the 6TiSCH protocol stack, (2) periodic on-board sensors sampling and (3) harvesting capabilities, in this case, used to support a primary battery.

### 6.1. 6TiSCH Network Power Characterization

A wireless industrial network such as 6TiSCH exploits the concept of TSCH, where time is divided in time slots grouped in a structure referred as Slotframe. For each time slot a node can use any of the *N* available frequencies leading to *N* cells per time slot. A network schedule determines the communication actions to be taken by the device at each these cells in the Slotframe. A node, in a particular cell, can transmit (TX), receive (RX) or transmit and if nothing to send, receive (TX/RX). The cell can be dedicated by a device or shared (contention) with other devices in the network.

TSCH has proven to enable ultra-low-power communications [18] thanks to tight synchronization, minimization of guard times, and the fact that nodes can be in sleep mode in the inactive cells. In this section we look into the energy consumed by the I3Mote when running a simple 6TiSCH network.

In a TSCH network, Slotframes repeat over time. The energy characterization is based on profiling the energy consumption on each of those slots and computing the overall energy consumption for each Slotframe. The average power can be obtained by dividing the total energy by the Slotframe period. Then, applying the model is a matter of counting the number of slots of each type and calculating the energy consumed by every one of the slots (see [24] for further details).

**Figure 9:**
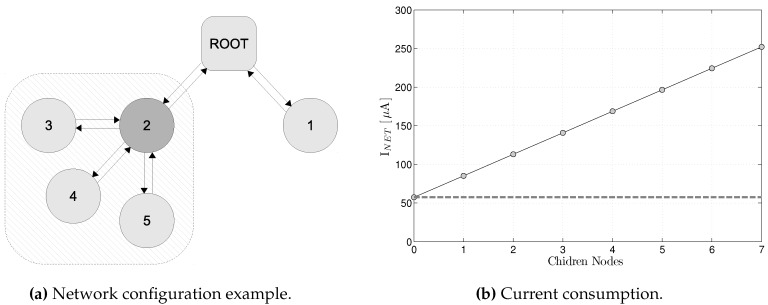
Power consumption as a function of the number of child nodes. On the left, the figure shows a possible configuration in which node 2, in addition to its own traffic, retransmits the traffic of 3 additional children nodes. The figure on the right shows the increment in energy consumption caused by the additional children.

Several experiments have been conducted in the simple network configuration depicted in Figure 9a. Table 2 presents the charge drained by the nodes when executing different types of slots. From this values, in Table 3 we have derived a simple model were we present the average energy consumption as a function of the active cells in the schedule for the different type of cells.

We consider different periodicity of actions, i.e., sending Enhanced Beacons happens once every 4 Slotframes while data traffic is scheduled to happen in every Slotframe. The Slotframe size of 127 timeslots and a default timeslot duration of 10 ms, with 10 s data duty cycle. As presented in Table 3, the average current consumption of this static setting is estimated by Equation (1):

(1) $$I_{NET}=27.9\;n+57.4\;\;\left[\mu A\right]$$

with *n* the number of child nodes (Figure 9 shows this equation graphically). For example, the energy consumption of Node-2 in Figure 9a, with 1 shared slot dedicated for join traffic and enhanced beacons [25] and with 4 additional active slots to send its own data and to relay traffic from 3 child nodes, is approximately 169 µA.

### 6.2. Sensors

The I3Mote integrates several low-power sensors including ambient light, magnetic sensor, humidity, pressure, accelerometer, magnetometer, object temperature and ambient temperature.

The current drained by the acquisition block can reasonably be approximated by a weighted average of the sleep and the active state of the sensors over the period *T*, according to Equation (2) (see [26] for a detailed description):

(2) $$I_{\mathrm{SENSOR}}=\frac{I_{\mathrm{ACTIVE}}\cdot T_{S}+I_{\mathrm{SLEEP}}\cdot \left(T-T_{S}\right)}{T}.$$

**Figure 10:**
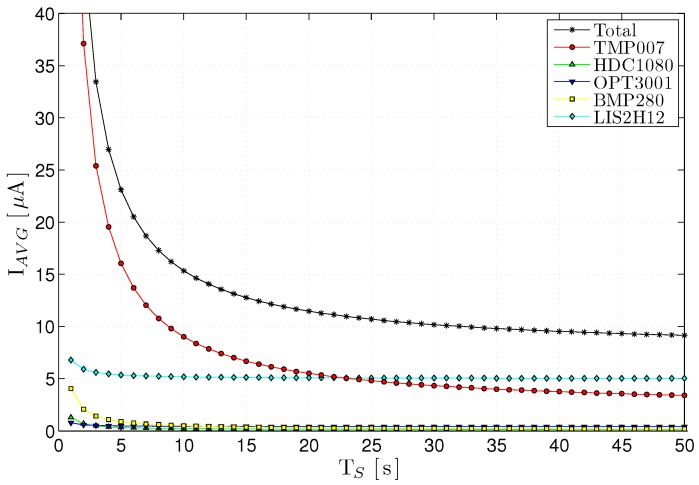
Power consumption of the I3Mote on-board sensors vs sampling rate. The average current consumption reaches an asymptotic minimum (below 10 µA) when the sampling interval is longer than 30 s.

Table 4 summarizes the consumption of the different sensors, both in the active state $I_{\mathrm{ACTIVE}}$ and in the low-power state $I_{\mathrm{SLEEP}}$, along with the typical time to read a sample $T_{S}$. Based on these values, Figure 10 shows the combined consumption of the sensor block for different sampling periods *T*, obtained from Equation (2).

### 6.3. System Considerations

Low-energy systems spend most of their time sleeping. The I3Mote has been specifically designed for minimal power in the sleep state. First, the two main cores (MSP432 and CC2650) are among the lowest energy consumption processors in the market. In their respective deep-sleep mode, the combined consumption is less than 1 µA. Second, special care has been taken in the selection of the components of the power supply (see Figure 4). With all DC sources in operation, the total quiescent consumption of the power subsystem is less than 6 µA. Finally, as we have seen in the previous section, the combined sleep current of the on-board sensors is approximately 7.5 µA. Taken together, the I3Mote quiescent current has been measured below 15 µA (with the microcontrollers in deep-sleep mode), a remarkable value for a system with almost 20 active components.

In order to test the I3Mote under actual operating conditions, a long-term experiment has been carried out. In this experimental configuration, an I3Mote “Engineering” (see Figure 2), acting as a DagRoot node, is connected to a Linux gateway embedded into a BeagleBone Black board. The BBB acting as 6LBR, handles all remaining 6TiSCH management tasks (joining, provisioning, topology and scheduling), a CoAP Client Module and a simple web server. In turn, the I3Mote “Tiny”, acting as a child node, connects directly to the root (one-hop). The embedded application is configured so that each *T* = 10 s reads all the on-board sensors, reporting the values acquired to the gateway.

**Figure 11:**
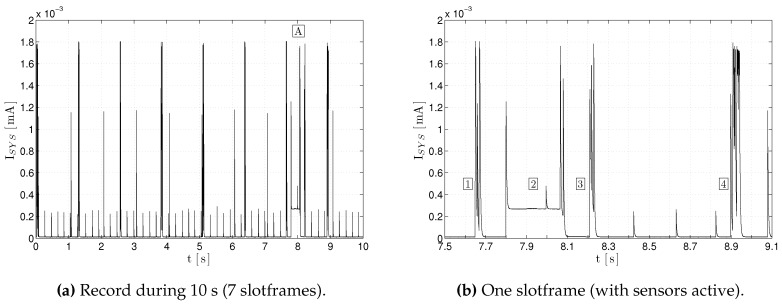
Current trace for a I3Mote node running the 6TiSCH stack.

Figure 11a shows a record of 10 s containing 7 slotframes (1.27 s each). Sensors are read and reported to the GW during the slotframe tagged as “A”. In detail, Figure 11b shows a zoom of the slotframe “A”. The slotframe is delimited by two Enhanced Beacons, labeled as “1” and “4”; the label “2” corresponds to the sensors reading (active state of the sensors) and the label “3” to the radio transmission of the acquired values.

In this experiment, the average current of the entire system drained from the battery is ${\bar{I}}_{BAT}$ = 103 µA, with 79.9% efficiency on the DC/DC stage.

### 6.4. Energy Harvesting

**Figure 12:**
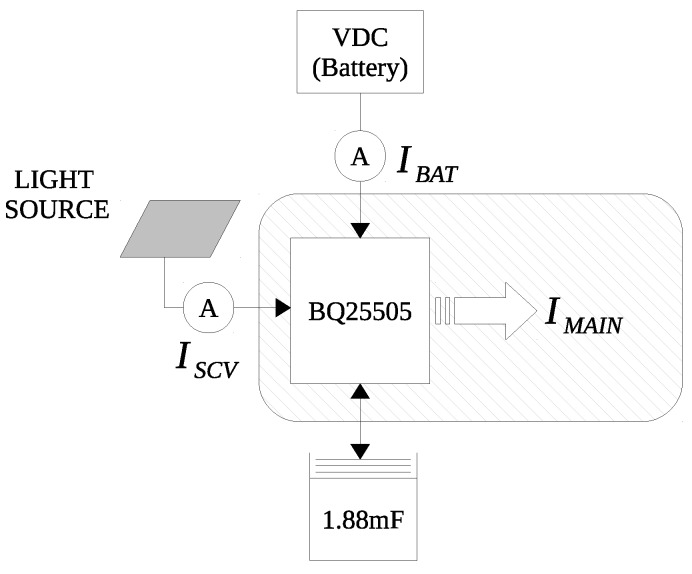
Experimental setup for the energy harvesting system

In this subsection, we describe how to maximize battery life by using the energy harvesting block. More specifically, we analyze the efficiency of energy harvesting with a solar panel designed for interior lights. We use energy harvesting as an assisting method for the primary battery, not considered as the main energy source to power the sensor node, even though perpetual energy harvesting is also possible when there is enough energy harvested from the environment. Figure 12 shows the I3Mote connection, the solar panel and the system testing diagram.

**Figure 13:**
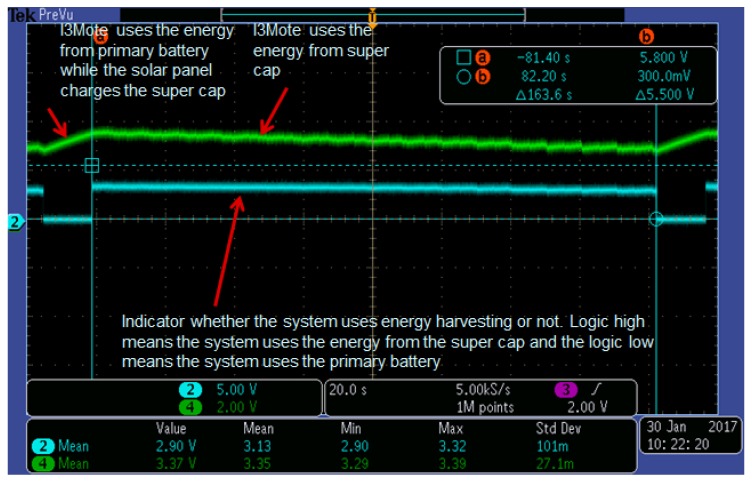
Energy harvesting charging and discharging graph. Ch4 shows the voltage at the super cap. Rising curve means the solar panel charges the super capacitor while the system uses the energy from the primary battery. Falling curve means the system uses the energy from the super capacitor while the energy is from the primary battery is not used.

If we assume that $V_{\mathrm{solar}}$ and $I_{\mathrm{solar}}$ are observed from the solar panel, and $V_{\mathrm{nominal}}$ is assumed at BQ25505 output, the charging time for the super cap is given by

(3) $$\Delta t_{\mathrm{charging}}=\frac{C\Delta V}{I_{\mathrm{supercap}}}=\frac{C\Delta V}{V_{\mathrm{solar}}I_{\mathrm{solar}}}V_{\mathrm{nominal}},$$

assuming 100% charging efficiency from the solar panel to the super-capacitor, where *C* is the capacitance of the super-capacitor, ΔV is the voltage difference at the super-capacitor for charging and discharging, and $V_{\mathrm{nominal}}$ is the nominal voltage at the super capacitor. The discharging time for the system from the super capacitor, (i.e., the system uses the super capacitor, not the primary battery), is given by

(4) $$\Delta t_{\mathrm{discharging}}=\frac{C\Delta V}{V_{\mathrm{system}}I_{\mathrm{system}}-V_{\mathrm{solar}}I_{\mathrm{solar}}}V_{\mathrm{nominal}}$$

where $V_{\mathrm{system}}$ and $I_{\mathrm{system}}$ are the system voltage and the system current consumption. Therefore, the effective time efficiency is defined by

(5) $$Eff≜\max\left(\frac{\Delta t_{\mathrm{discharging}}}{\Delta t_{\mathrm{charging}}+\Delta t_{\mathrm{discharging}}},1\right)=\max\left(\frac{V_{\mathrm{solar}}I_{\mathrm{solar}}}{V_{\mathrm{system}}I_{\mathrm{system}}},1\right)$$

and the amount of the primary battery life extension is given by

(6) $$\frac{1}{1-Eff}=\left\{\begin{array}{cc} \frac{V_{\mathrm{system}}I_{\mathrm{system}}-V_{\mathrm{solar}}I_{\mathrm{solar}}}{}, & \mathrm{if}\;V_{\mathrm{system}}I_{\mathrm{system}}>V_{\mathrm{solar}}I_{\mathrm{solar}}\\ \infty , & \mathrm{otherwise} \end{array}\right.$$

where ∞ means the system fully runs from the energy harvesting. Figure 13 shows the energy harvesting efficiency. In our indoor light environment under 300 lux light we can observe 92% of the efficiency, which corresponds to 13X battery life extension of the primary battery. For example, with two AAA batteries, our system can run for 1.36 years over a 6TiSCH network with 10 s periodic duty cycling and with the energy harvesting under 300 lux indoor light, it can run for 17.54 years.

**Figure 14:**
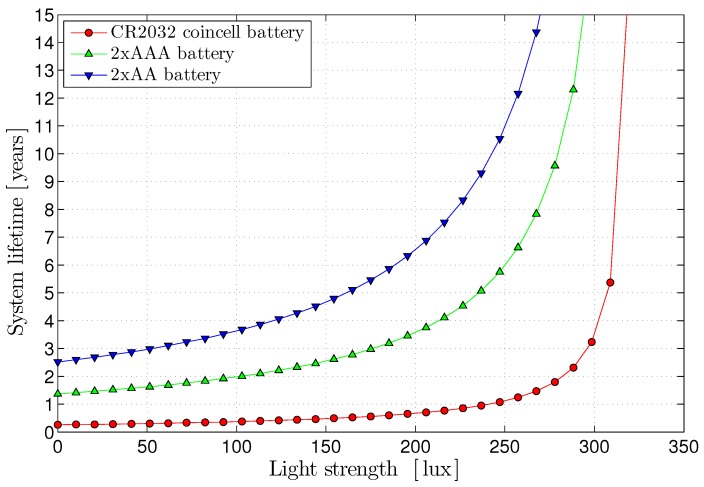
Battery life extension for various types of batteries under the indoor light strength in lux.

Figure 14 shows the system life time for various battery types. With 316.2 lux indoor light, we can achieve alifetime of more than 10 years for the on-board coin-cell batteries.

## 7. Discussion

The use of wireless deterministic technologies such as 6TiSCH is gradually being understood and adopted by industries to complement existing sensing infrastructures (usually wired). Wireless communications provide lower operating costs by reducing wiring and driving needs. This results in simple, non-intrusive deployment, thereby reducing operational requirements and minimizing training efforts as networks are self-organizing. The increased demand for these technologies from an application perspective, the recent advances on low power radio and microncontrollers and the consolidation of low-power wireless standards targeting industrial requirements has accelerated the development of novel wireless-enabled products.

The I3Mote is positioned as a development platform between pure prototyping and the a final product. This aims to address an early demand of such a technology and enable software customization exploiting general-purpose functionalities of the designed device. With the I3Mote we facilitate product development promoting platform-based design as we believe that the bottom-line requirements for most of the use cases are similar. In this line, we have understood that support for a wide range of sensing devices, including legacy industrial buses, powerful computing capabilities for embedded data fusion and ultra-low-power wireless communication interfaces are key corner stones to support any future-proof industrial device.

The I3Mote ecosystem is designed to support quick industrial adoption in industrial automation and control scenarios possibly where industrial sensors need to be interfaced and data extracted reliably to the Internet. The device ecosystem aims to facilitate product development by providing license-free software, toolchain and examples. The open licensing of the software, hardware and tools contribute to the democratization of its design enabling industries to reuse most suitable parts to tailor the design in order to obtain cost-effective products.

The future direction we take will complement the ecosystem with a new I3Mote hardware, addressing other industrial demands such as serial buses with RS485, providing ModBus support and enabling longer range connectivity through subGhz radio interfaces.

## Figures and Tables

**Table 1:** Features Summary.

	Power (x)		Wireless 2.4 GHz (o)				Industrial Bus (+)				Sensors (*)		
Platform	Low Power	Energy Harvesting	802.15.1 (BLE)	802.15.4e (6TiSCH)	WirelessHART	WiFi	Ind. Ethernet	HART	CAN	RS-485	Onboard Sensors	External Sensors	Mechanical Format
SensorTAG	x	-	o	o	-	-	-	-	-	-	10	-	Fully Embedded
Bosch XDK	x	-	o	-	-	o	-	-	-	-	8	-	Fully Embedded
Linear/Dust	x	-	-	-	o	-	-	-	-	-	1	-	Module
OpenMote	x	-	-	o	-	-	-	-	-	-	1	-	Dev. Kit
OpenPicus	x	-	-	-	-	o	+	-	-	-	-	-	Expansion Card
Libelium	-	-	o	o	-	o	+	-	+	+	-	*	Modular
MongOH	-	-	o	-	-	o	+	-	+	+	2	*	Modular
I3Mote	x	x	o	o	-	-	-	+	-	-	6	*	Fully Embedded

**Table 2:** CC2650 6TiSCH consumption.

Action	Charge [μC]
Beacon RX (84 bytes Broadcast Packet—no ACK)	34.62
Beacon TX (84 bytes Broadcast Packet—no ACK)	32.92
TX (102 bytes Unicast Packet, 25 bytes ACK )	48.67
TX (no packet transmission)	2.26
RX (102 bytes Unicast Packet, 25 bytes ACK )	50.6
RX (no packet reception)	23.98

**Table 3:** CC2650 6TiSCH consumption, with 10 s data duty cycle.

Link type	Number of links	Wake-up interval	I${}_{6\mathrm{TiSCH}}$ [μA]
Beacon RX	1	4	6.8
Beacon TX	1	4	6.5
Idle listening in Shared Slot	1	1	18.9
Dedicated TX	n+1	1	6.35*n* + 6.33
Dedicated RX	n+1	1	21.5*n* + 18.9
Total			27.9*n* + 57.4

**Table 4:** On-board sensors power consumption.

Sensor	I${}_{\mathrm{ACTIVE}}$ [μA]	I${}_{\mathrm{SLEEP}}$ [μA]	T${}_{S}$ [ms]	Configuration
TMP007	270	2	260	
HDC1080	190	0.1	6.2	Temperature Sample 11 bit; Humidity 8 bit
OP3001	3.7	0.4	100	Full Scale Range Lux
BMP208	720	0.1	5.5	1 Pressure + 1 Temperature Samples
LIS2H12	180	5	10	ODR=100 Hz
